# Brief version of the coping health inventory for parents (CHIP) among family caregivers of children with chronic diseases

**DOI:** 10.1186/s12955-020-01357-5

**Published:** 2020-04-19

**Authors:** Filiberto Toledano-Toledano, José Moral de la Rubia, Laurie D. McCubbin, Bridget Cauley, David Luna

**Affiliations:** 1 Unidad de investigación en Medicina Basada en Evidencias del Hospital Infantil de México Federico Gómez National Institute of Health, Dr. Márquez 162, Doctores, Cuauhtémoc, 06720 México City, Mexico; 2 Facultad de Psicología, Universidad Autónoma de Nuevo León, Dr. Carlos Canseco, 110, Esq. Dr. Aguirre Pequeño, Col. Mitras Centro, 64460 Monterrey, Mexico; 3 College of Education and Human Development, University of Louisville, 1905S1st St, Louisville, KY 40208 USA; 4 Comisión Nacional de Arbitraje Médico, Mitla No. 250-10° Floor, esq. Axis 5 South (Eugenia). Col. Narvarte, 03020 Benito Juárez, Mexico City, Mexico

**Keywords:** Coping, Anxiety, Depression, Psychometrics, Family caregivers, Mexico

## Abstract

**Background:**

The Coping Health Inventory for Parents (CHIP) has demonstrated good psychometric properties in several language forms and has been used to assess the coping behaviors of families facing disease. However, the CHIP has not been validated in Mexico among families of children with chronic conditions, where it could be useful for research and intervention. The objectives of this instrumental study were to obtain a version of the CHIP for the Spanish language in Mexico, establish the factor structure of the Mexican version of the CHIP, probe its internal consistency reliability, and assess its concurrent construct validity.

**Methods:**

A nonprobability sample of 405 family caregivers of children with chronic diseases responded to a battery of measurement instruments that included the CHIP, the Beck Anxiety Inventory, and the Beck Depression Inventory. The sample was randomly divided into two parts. In one subsample (190 participants), an exploratory factor analysis was performed using a principal component analysis and oblique rotation. In the second subsample (215 participants), a confirmatory factor analysis was performed using maximum likelihood estimation.

**Results:**

The scale was reduced to 16 items (CHIP-16) with factorial loads greater than .50. The empirical criteria used to determine the number of factors converged on the following five factors: belief and trust (McDonald ω = .85), spouse/partner relationship (ω = .79), home care (ω = .77), family involvement (ω = .75), and security/stability (ω = .79). The overall internal consistency was good (ω = .88). The five-factor model showed acceptable fit indices and high parsimony. The mean CHIP-16 scores and the Spouse/partner relationship scores among the caregivers with anxiety were greater than those among the caregivers without anxiety. The mean home-care scores among the women were greater than those among men.

**Conclusions:**

The 16-item version of the CHIP showed good internal consistency and construct validity; thus, the CHIP-16 is a useful instrument for measuring and assessing coping in family caregivers of children with chronic diseases.

## Background

Pediatric chronic diseases represent a central event constituting a major challenge for families; these diseases have physical, psychological, socioeconomic, and behavioral effects on patients and their family caregivers that translate into vulnerability and decreased quality of life and family functioning [1]. In the research literature, a family caregiver is defined as a person who has a significant emotional bond with the patient; this caregiver is a family member who is a part of the patient’s family life cycle, offers emotional-expressive, instrumental, and tangible support, and provides assistance and comprehensive care during the chronic illness, acute illness, or disability of a child, adult, or elderly person [2]. The diagnosis of a chronic pediatric disease is an event that affects not only the patients but also their nuclear family; in this context of adversity, parents need to acquire skills that allow them to perform the medical and personal care that the pediatric patient with a chronic disease requires [3]. An adaptive coping mechanism during chronic disease allows parents to avoid psychopathological problems that could have negative psychosocial consequences on patient care and interactions with medical service providers [4].

In the context of chronic pediatric diseases, parental problem-focused and socially supported coping strategies are factors protecting their health [3] and contributing to the psychological well-being and health of the child [5]. In addition, empirical evidence has shown that the use of active coping strategies is related to an increase in well-being, reduction in perceived stress, and a process of positive adaptation despite the loss of health; it involves the development of vitality and skills to overcome the negative effects of adversity, risks, and vulnerability caused by disease [6]. Because coping behaviors have a positive effect on both children’s and caregivers’ well-being, a compelling need exists to establish meaningful ways to assess how parental caregivers respond to the challenges of children with chronic diseases [7].

Although several instruments assessing coping strategies have been validated [8–10], the Coping Health Inventory for Parents (CHIP) [11] has been the most widely used in the context of pediatric chronic diseases [3]. The origin of this instrument lies in the resilience model for family stress, adjustment, and adaptation [12], which explains how family members faced with adverse conditions adjust and adapt using the resources they have available (e.g., coping strategies). The CHIP construction was guided by a compilation of behavioral items used in prior studies investigating family coping responses to stress [13, 14]; then, additional behavioral items focusing on the following aspects were developed: a) social support theory [15, 16]; b) family stress theory [17], which underscores the resources and perceptions that a family employs in the management of a stressor event; c) theories of individual psychology of coping [10, 18]; and d) family health care support [17]. Additionally, CHIP development was influenced by a hierarchical approach to the organization of behavior. In the application of this approach, three levels of coping were defined as follows: 1) coping behaviors described by each item on the inventory, 2) coping patterns involving combinations of specific coping behaviors, and 3) factors and overall coping encompassing different coping patterns [17].

Given the usefulness of this tool in assessing parental coping, the CHIP has been validated in languages other than English and various populations [3, 5, 19]. These CHIP validation studies and the characterization of parental coping strategies in other populations facilitate the design and evaluation of culturally relevant interventions and enable the incorporation of instruction to various parents of pediatric patients.

In the existing studies exploring coping, the greatest differences between the sexes appear to be the search for support from the family and spouse (socially supported coping), which is more frequently used by women [20, 21], and home care, which is more frequently performed by women [22]. However, the impact of sex on coping is minimal in these studies.

Despite the usefulness and importance of a coping measurement instrument for parents, to date, no version of the CHIP validated in Spanish with an accompanying report of its psychometric properties has been reported. This omission constitutes a gap in the research in this field because no empirical evidence of the validity and reliability of the CHIP in Spanish-speaking contexts and populations exists, and evidence regarding its methodological importance in assessing the parental caregiver coping strategies during a child’s chronic disease in this context is lacking. Therefore, to contribute to the theoretical approaches of coping and provide empirical evidence concerning the CHIP in different populations and cultures, the objectives of this study were to 1) obtain a Spanish language version of the CHIP for use in Mexico; 2) test the original model of three correlated factors; 3) verify the internal consistency reliability and convergent validity of each factor and the discriminant validity among the factors; 4) explore the factor structure of the CHIP’s 45 items to determine how many factors to retain and the configuration of those factors in the case the original model shows poor properties of fit, reliability and validity; 5) test the factor model derived from an exploratory factor analysis (EFA) in an independent sample; and 6) provide evidence of concurrent construct validity by verifying whether caregivers with anxiety or depression obtain lower means than caregivers without anxiety or depression and whether women use family support and involvement with home care more often than men.

## Methods

### Ethical considerations

This study is a part of the Research Project HIM/2015/017/SSA.1207 “*Effects of mindfulness training on the psychological distress and quality of life of the family caregiver,*” which was approved by the Research, Ethics, and Biosafety Commissions of the Hospital Infantil de México Federico Gómez National Institute of Health, in Mexico City. While conducting this study, the ethical rules and considerations for research with humans currently enforced in Mexico [23] and those outlined by the American Psychological Association [24] were followed. All family caregivers were informed of the objectives and scope of the research and their rights according to the Helsinki Declaration [25]. The caregivers who agreed to participate in the study signed an informed consent letter. Participation in this study was voluntary and did not involve payment.

### Participants

This instrumental study [26] used a cross-sectional design in which a nonprobabilistic sampling technique was used to recruit a sample of 405 voluntary participants who were family caregivers of pediatric patients with chronic diseases at the Hospital Infantil de México Federico Gómez. The sample size was calculated to meet the criterion of recruiting at least five participants per item of the instrument to be validated [27]. The inclusion criteria included a family caregiver who played a parenting role, had a child with a chronic disease that required highly specialized hospital treatment, and provided informed consent. The exclusion criteria included being illiterate or refusing to voluntarily participate in the study. Chronic disease is defined as a medical condition that typically lasts 3 months or longer and may worsen over time. The most common types are cancer, heart disease, and diabetes.

### Instruments

1. *A Sociodemographic variables questionnaire (Q-SV) for research on family caregivers of children with chronic disease.* This instrument comprises 20 items that measure individual, familial, and caregiver factors such as age, sex, schooling, and marital status of the caregiver; the type, life cycle, and income of the family. In addition, this instrument includes the child’s sex, age, diagnosis, and length of hospitalization [28].
2. *The Coping Health Inventory for Parents (CHIP)* [11]. The original CHIP instrument comprises 45 self-report items with five response options. The items are grouped into the following three factors: 1) maintaining family integration, cooperation, and an optimistic definition of the situation (19 items); 2) maintaining social support, self-esteem, and psychological stability (18 items); and 3) understanding the medical situation by communicating with other parents and consulting with medical staff (8 items). The overall reliability of the CHIP was good (α = .89), and each of its three factors was acceptable, with alphas ranging from 71 to 79 [29].
3. *The Beck Anxiety Inventory (BAI)* [30] has been adapted to a Mexican population [31]. In the BAI, caregivers are requested to rate the presence and severity of anxiety symptoms to discriminate between anxiety and depression. The BAI comprises 21 items with four response options used to rate the severity of the symptom. The items are divided into the following four factors: subjective, neurophysiological, autonomic, and panic (fear that the worst scenario will occur, nervousness, and fear of dying). A cut-off score of 10 for the BAI total score is used to define a case with anxiety symptoms [31]. The overall reliability of the BAI was good (α = .84) in a Mexican validation study [31]. In the present study, the overall reliability was excellent (α = .92).
4. *The Beck Depression Inventory (BDI-II)* [32] has also been adapted to a Mexican population [2]. The BDI-II evaluates the magnitude of individual depression. The BDI-II comprises 21 items that evaluate various symptoms, such as pessimism, crying, and changes in appetite. Each item has four response options used to rate the severity of the symptom, and the items are divided into the following two factors: cognitive-affective and somatic symptoms of depression. The BDI has a cut-off score of 14 for defining a case with symptoms of depression [2]. In this study, the alpha coefficient was good (α = .91).

### Procedures

The specific process used for the cross-cultural adaptation of the CHIP instrument was based on the back-translation method [33]. Initially, the instrument was translated into Spanish. Then, the Spanish translation was retranslated into English by independent translators who did not know the objectives of the study. Subsequently, a group of specialists reviewed the disagreements between both translations. These discrepancies were solved by considering the semantic content of the original CHIP and the cultural context of the Spanish language in Mexico. Finally, the CHIP version translated into Spanish was applied to 40 family caregivers voluntarily recruited at the Hospital Infantil de México Federico Gomez National Institute of Health. These caregivers were asked about the clarity and understandability of the items, and the items causing confusion were modified. The caregivers evaluated the items on a three-point scale, and free responses were allowed such that the participants could explain the confusing or unclear aspects of each item.

The data collection was performed by trained personnel at the Evidence Based Medicine Research Unit of the National Institute of Health under the direction of the first author of this study. The data collection process lasted approximately 5 months and occurred in the rooms of the hospitalized children and the waiting rooms of the different medical services of the institution. Consenting caregivers were given instructions and completed the questionnaires independently while visiting their children’s hospital. The battery of tests was individually administered.

### Data analysis

First, the original model was tested using a confirmatory factor analysis (CFA). The discrepancy function was optimized by using maximum likelihood (ML) estimation. The following eight goodness-of-fit indices were calculated: the probability of the likelihood-ratio chi-square statistic (p of χ^2^), relative likelihood-ratio chi-square statistic (χ^2^/df), Jöreskog and Sörbom’s Goodness-of-Fit Index (GFI), Adjusted Goodness-of-Fit Index (AGFI), Incremental Fit Index (IFI), Tucker-Lewis Index (TLI), Bentler’s Comparative Fit Index (CFI), and Root Mean Square Error of Approximation (RMSEA). Parsimony was evaluated using James, Mulaik and Brett’s Parsimony Ratio (PR) and the parsimonious indices of GFI and CFI. The cut-off values for the interpretation of the goodness-of-fit indices in terms of close or acceptable fit and the parsimonious indices are shown in Table 1 [34].

**Table 1:** Fit and parsimony indices of the 5-factor model with 16 items

Index		Cut-off values		Models	
		*Close*	*Acceptable*	3F-45 items	5F-16 items
Fit	χ^2^	*p* > .05	*p* > .01	χ^2^ [942, *N* = 405] = 3055.942, *p* < .001	χ^2^ [93, *N* = 215] = 152.504, *p* < .001
	χ^2^/df	< 2	< 3	3.244	1.640
	GFI	> .95	> .90	.706	.914
	AGFI	> .90	> .85	.677	.874
	IFI	> .95	> .90	.644	.923
	TLI	> .95	> .90	.623	.896
	CFI	> .95	> .90	.642	.920
	RMSEA (90% CI)	<.05	< .08	.075 (.072, .077)	.057 (.040, .073)
Parsimony	PR	> .75	> .50	.952	.775
	PGFI	> .70	> .50	.643	.625
	PCFI	> .80	> .60	.610	.713

*N* = 215. Method: Maximum likelihood estimation. Indices of model fit. χ^2^ = Minimum Value of Discrepancy or Likelihood-Ratio Chi-Square Statistic, χ^2^/df = Relative Likelihood-Ratio Chi-Square Statistic, GFI = Goodness-of-Fit index, AGFI = Adjusted Goodness-of-Fit Index, IFI = Incremental Fit Index, TLI = Tucker-Lewis Index, CFI = Comparative Fit Index, and RMSEA = Root Mean Square Error of Approximation. PR = Parsimony Ratio, PGFI = Parsimonious Goodness-of-Fit index, and PCFI = Parsimonious Comparative Fit Index

The convergent validity of a factor can be defined as the proportion of variance in the observed variable (indicator) explained by an unobserved variable (factor) greater than the proportion explained by non-attributable factors [35]. The average variance extracted (AVE) was used as an index of convergent validity; an AVE value greater than .50 indicates that a factor has convergent validity [36]. However, a factor has discriminant validity with respect to the other factors within the factorial model when the proportion of variance in its items explained by the factor is greater than the proportion explained by the other factors related to that factor. A shared variance between two factors (a squared correlation between these two factors) less than the AVE of each factor and less than two-thirds suggests discriminant validity between these factors [37]. The internal consistency reliability, which is defined as the proportion of variance without error that appears in the variance of the factor or test scores, was calculated using McDonald’s coefficient omega or composite reliability coefficient. This coefficient overcomes the assumption that the items are tau-equivalent as required by Cronbach’s alpha coefficient; in addition, this coefficient is considered the best estimator of internal consistency reliability [38, 39]. Internal consistency reliability values below .50 are considered unacceptable; values ranging from .50 to .59 are considered poor; values ranging from .60 to .69 are questionable; values ranging from .70 to .79 are acceptable; values ranging from .80 to .89 are good; and values ranging from .90 to 1 are excellent [39].

A new model was explored due to the poor properties of the fit of the model, the convergent validity of each factor and the discriminant validity between the factors. In addition, cultural and contextual differences could justify the exploration of a new model [33]. Therefore, the total sample was randomly divided into the following two parts: one part was used for an EFA, and the other part was used for a CFA. Horn’s parallel analysis [40], optimal coordinates [41], Ruscio, Roche’s comparison data [42], Velicer’s Minimum Average Partial (MAP) test [43] and Kaiser’s criterion or an eigenvalue greater than one [44] were used to establish the number of factors. The convergence of these criteria was sought [45].

The EFA was performed based on a Pearson’s correlation matrix using the principal component extraction method and direct oblimin rotation. The criteria used to eliminate items included a factorial weight less than .50 (in the structural matrix) and extracted commonalities less than .25 based on the criterion of a large size for a standardized regression weight or a correlation coefficient [36]. This approach was used to obtain strong indicators to achieve convergent validity in the factors [36, 46]. The AVE and composite reliability of each factor were calculated from the matrix structure. Subsequently, a CFA of the variance-covariance matrix was performed to test the factor model obtained via the EFA using ML.

Finally, t-tests comparing the means of the two independent groups and Cohen’s *d* computing the effect size were used to determine the concurrent construct validity of the scale. The data were analyzed using SPSS v.24, IBM Inc., Chicago, USA (EFA and t-test), Excel 2013 (AVE, McDonald’s coefficient omega and Cohen’s *d*), SPSS R Menu 2.4 (parallel analysis, comparison data, optimal coordinates, and MAP), and AMOS 22 (CFA).

## Results

### Description of the sample

Table 2 presents the sociodemographic characteristics of the parent sample, and Table 3 provides the demographic and clinical characteristics of the children.

**Table 2:** Descriptive analysis of the sociodemographic and clinical characteristics of the parents

Characteristics	*n* (%)	*M* (*SD*)
*Age*		31.93 (7.99)
*Sex*		
Women	320 (79)	
Men	85 (21)	
*Level of schooling*		
High school	156 (38.5)	
Elementary	145 (35.8)	
Technical training	75 (17.5)	
No formal education	17 (4.2)	
Bachelor’s degree	16 (4)	
*Occupation*		
Housewife	303 (74.8)	
Student	44 (10.9)	
Employee	40 (9.9)	
Businessman/businesswoman	14 (3.5)	
Professional	4 (1)	
*Marital status*		
Married	184 (45.4)	
Cohabitating	180 (44.4)	
Separated	41 (10.1)	
*Type of family*		
Nuclear	223 (55.1)	
Extended	111 (27.4)	
Semi-extended	55 (13.6)	
Single-parent	16 (4)	
*Family life cycle*		
Family with school-aged children or adolescents	236 (58.3)	
Family with small children	144 (35.6)	
Family with adult children	25 (6.2)	
*Hours of care for the patient*		
Between 1 and 8 h per day	323 (79.8)	
Beteen 9 and 12 h per day	50 (12.3)	
More than 13 h per day	35 (7.9)	
*Monthly family income*		
< 456 USD	281 (69.38)	
[456, 826) USD	120 (29.62)	
[826, 1620] USD	4 (0.98)

**Table 3:** Descriptive analysis of the sociodemographic characteristics of the children

Characteristics	*n* (%)	*M* (*SD*)
*Sex*		
Boys	212 (52.3)	
Girls	193 (47.7)	
*Age*		6.69 (4.28)
*Time of hospitalization*		
Between 0 and 3 months	373 (92.09)	
6 months	26 (6.4)	
1 year	6 (1.5)	
*Diagnosis*		
Oncological patients	307 (75.2)	
Abnormal blood flow due to congenital heart defect	31 (7.6)	
Nephrotic syndrome	19 (4.7)	
End-stage renal disease	15 (3.7)	
Tricuspid atresia	10 (2.4)	
Asthma	10 (2.4)	
Down syndrome	8 (2)	
HIV/SIDA	6 (1.5)	
Cystic Fibrosis	2 (0.5)	

### Cross-cultural adaptation of the CHIP

Some discrepancies occurred in five phrases between the translation from English to Spanish and the one from Spanish to English (items 2, 5, 15, 18, and 26). A group of experts (two health psychologists with knowledge about the objectives of study and the conceptual frame of the CHIP and two native linguists) resolved these discrepancies by considering the semantic content of the original CHIP and the cultural context of the Spanish language in Mexico. When the clarity and understandability of the items were evaluated in the sample of 40 family caregivers, some confusing aspects were identified in two phrases (items 5 and 15); this confusion was corrected by the four experts based on suggestions given by the participants.

### Testing the original model by a CFA

**Fig. 1:**
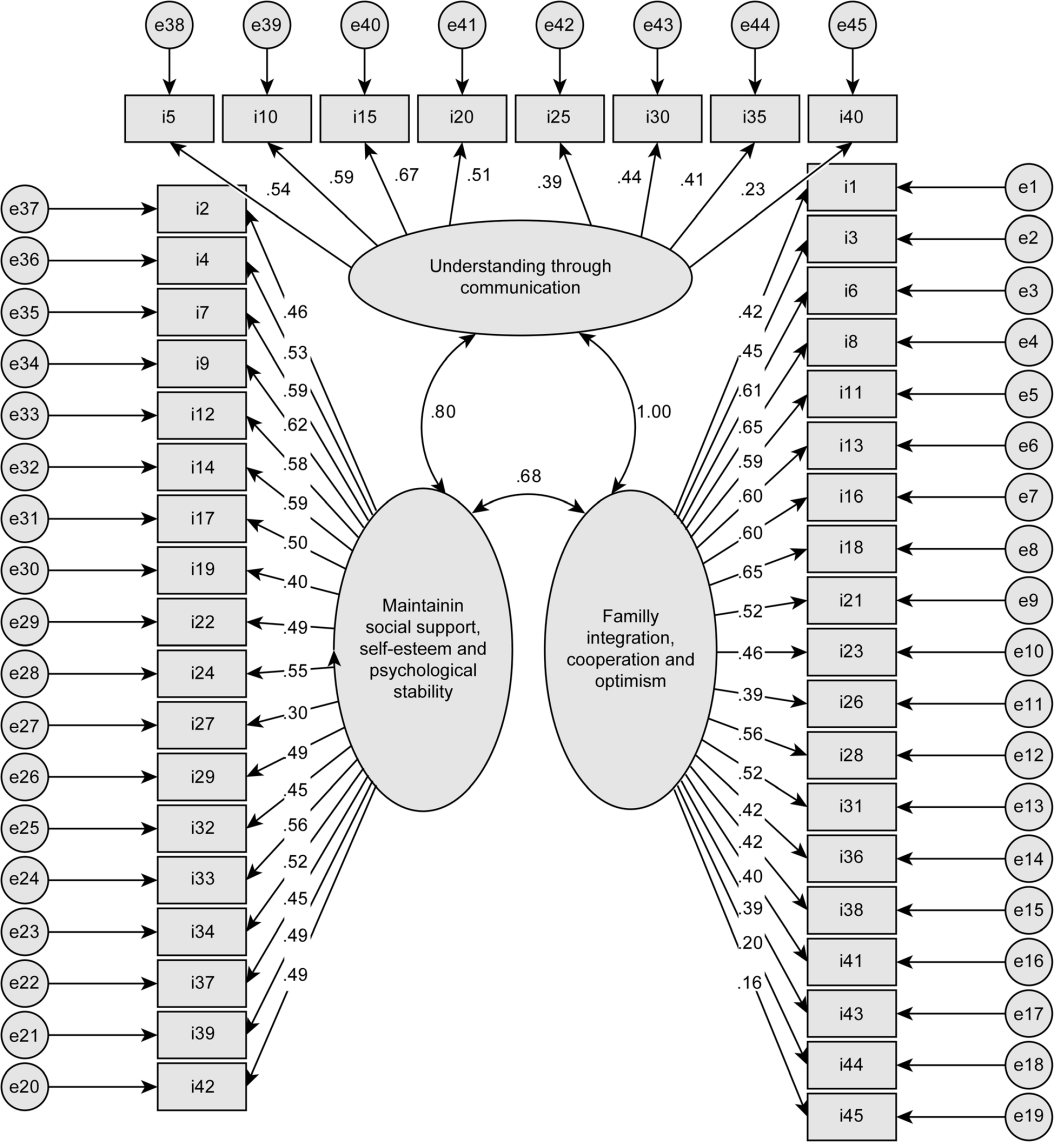
Original model of three correlated factors computed by the maximum likelihood in a total sample of 405 participants

The correlated three-factor model was tested in the total sample. The solution converged in 13 iterations and was admissible (Fig. 1). All parameters were significant. The first factor of family integration, cooperation and optimism with 19 indicators presented good composite reliability (ω = .85) but lacked convergent validity (AVE = .24) and discriminant validity (r^2^ = .46 with F2 and .99 with F3). The second factor of maintaining social support, self-esteem and psychological stability with 18 indicators also showed good composite reliability (ω = .86) but again lacked convergent validity (AVE = .26) and discriminant validity (r^2^ = .46 with F1 and .64 with F3). Similar to the two previous factors, the third factor of comprehension through communication had acceptable composite reliability (ω = .70) but lacked convergent validity (AVE = .24) and discriminant validity. Despite the very high parsimony of the model, the fit indices were poor, except for the RMSEA, which exhibited an acceptable fit value (Table 1). Due to these unfavorable results, it was decided to explore the factor structure of the CHIP. The total sample of 405 participants was divided into the following two subsamples: one subsample of 190 participants was used to explore the new model through an EFA, and another subsample of 215 participants was used to test the new model through a CFA.

### Exploration of the factor structure of the CHIP

**Fig. 2:**
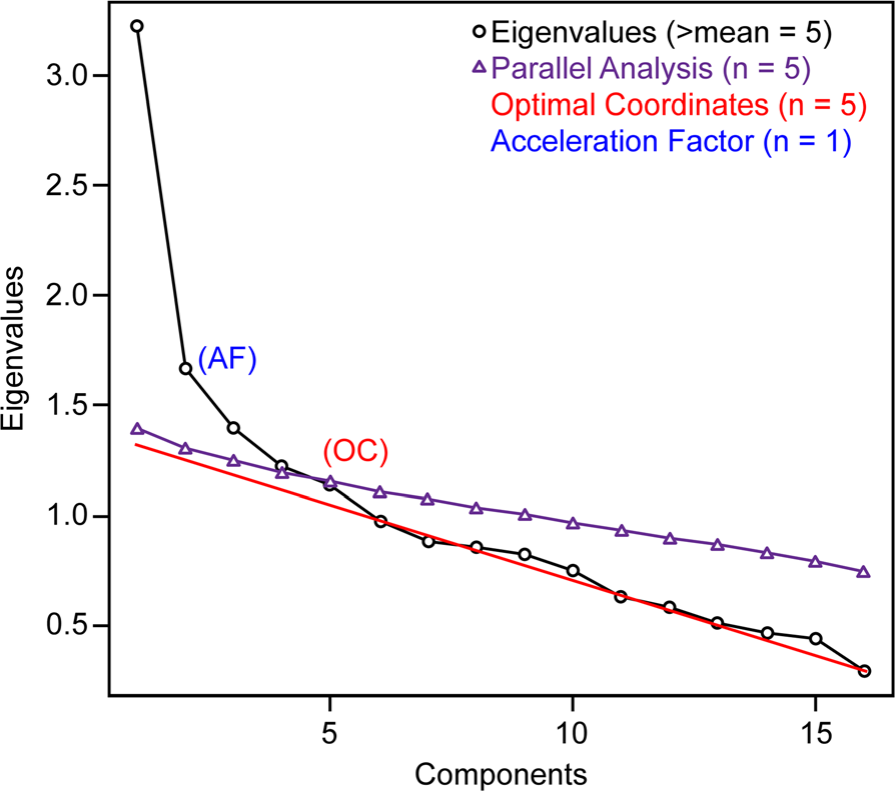
Parallel analysis applied to the final 16 selected items in a subsample of 190 participants

**Fig. 3:**
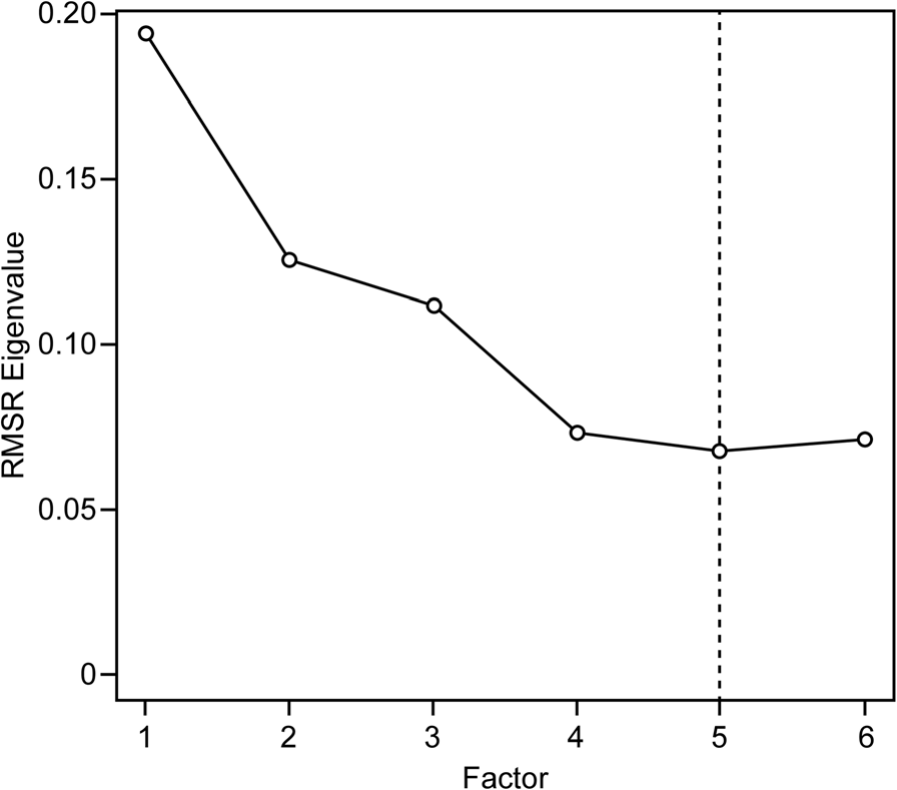
Data comparison analysis applied to the final 16 selected items in a subsample of 190 participants

The subsample of 190 randomly selected respondents was used for an EFA. The Kaiser-Meyer-Olkin Measure (KMO = .81) and Bartlett’s Test of Sphericity (χ^2^[990, *N* = 190] = 7382.47, *p* < .01) confirmed the legitimacy of the EFA and the presence of underlying factors among the 45 initial items. Thirteen eigenvalues were greater than one. Horn’s parallel analysis and optimal coordinates indicated eight factors. Ruscio and Roche’s comparison data, squared MAP and 4th power MAP coincided in five factors. In successive steps, the original CHIP with 45 items was reduced to 16 items. In total, 29 items were excluded until a convergence of criteria was reached (Horn’s parallel, optimal coordinates, Ruscio and Roche’s comparison data, squared MAP and eigenvalues greater than one) for five factors (Figs. 2 and 3), and all retained items had loads greater than .50 in one factor within the matrix structure (Table 4).

**Table 4:** Structural matrix of the five factors with the 16 selected items

Items	F1	F2	F3	F4	F5
15. Communicating with a doctor regarding my concerns about my child with a medical condition.	**.790**	.125	.265	−.257	.267
17. Engaging in relationships and friendships that help me feel important and appreciated.	**.750**	.075	.220	−.160	.192
16. Believing that the medical center/hospital considers my family’s best interests.	**.746**	.005	.087	−.220	.302
18. Believing in God.	**.734**	.283	.140	−.262	.282
5. Communicating with the medical staff (nurses, social worker, etc.) when we visit the medical center.	**.630**	.261	.119	−.380	.187
35. Ensuring that the prescribed medical treatments for my child are performed at home on a daily basis.	.197	**.849**	.100	−.101	.106
38. Investing myself in my children.	.161	**.728**	.122	−.434	.149
36. Building a closer relationship with my spouse.	.063	**.672**	.270	−.171	.538
31. Encouraging my children with a medical condition to be more independent.	.490	**.517**	−.106	−.445	.149
26. Doing things with family relatives.	.157	.143	**.870**	−.047	.015
21. Doing things together as a family.	.451	.086	**.698**	−.290	.132
43. Having my child with a medical condition visit the clinic/hospital on a regular basis.	.295	.112	−.058	**−.783**	.140
41. Trying to maintain family stability.	.228	.289	.195	**− 766**	.147
39. Communicating with someone (other than a professional counselor/doctor) about how I feel.	.231	.203	.477	**−.552**	−.041
1. Communicating about personal feelings and concerns with my spouse.	.260	.214	.047	−.161	**.811**
3. Trusting my spouse to help support me and my child.	.438	.000	−.064	−.119	**.807**
NI	5	4	two	3	two
AVE	.536	.492	.622	.502	.654
ω	.852	.790	.765	.747	.791

*N* = 190. Extraction method: Principal component analysis. Rotation method: Oblimin with Kaiser normalization. The rotation converged in 10 iterations. *F1* Belief and trust, *F2* Spouse and children’s relationship, *F3* Home care, *F4* Family involvement, *F5* Security and stability, *NI* Number of items, *AVE* Average variance extracted, ω = McDonald’s coefficient omega or composite reliability

The mean AVE of these five factors was 56.6%; the overall composite reliability was good (ω = .86); and the mean shared variance between the factors was low (3.7%), ranging from .01 to .10.

Factor I, i.e., *beliefs/trust*, comprised five items regarding having trust in the medical center and God, communicating with medical staff, and having close relationships with people. The internal consistency of this factor was good (ω = .87) and had convergent validity (AVE = .54).

Factor II, i.e., *spousal/partner relationship*, comprised four items regarding communicating about personal feelings and concerns with a spouse/partner and trusting the spouse/partner to help support the caregiver and the caregiver’s children. With four indicators, the internal consistency of this factor was high (ω = .80), and the AVE was close to .50 (AVE = .49).

Factor III, i.e., *home care,* comprised two indicators regarding home care by ensuring that prescribed medical treatments for the children were conducted at home on a daily basis, investing time in the children, building a close relationship with the spouse/partner, and encouraging the chronically ill child. This factor had acceptable internal consistency (ω = .77) and convergent validity (AVE = .62).

Factor IV, i.e., *family involvement*, comprised three indicators regarding family involvement and engaging family members, shared activities with related family members, and shared activities as a family. This factor had acceptable internal consistency (ω = .75) and convergent validity (AVE = .50).

Factor V, i.e., *security and stability*, comprised the following two items: having a child seen at a clinic/hospital on a regular basis, communication with a person other than a professional counselor or doctor, and maintaining family stability. With two indicators, the internal consistency of this factor was acceptable (ω = .79), and this factor had convergent validity (AVE = .65).

The correlations among the five factors ranged from .09 to .32. Therefore, each factor showed discriminant validity. The shared variance of each factor with the other four factors was lower than each factor’s AVE and less than two-thirds. The highest shared variance was less than 4%.

The correspondence between the five factors defined by the 16 items selected from the results of the EFA (CHIP-16) and the three factors defined by the 45 original items of the CHIP is shown in Table 5.

**Table 5:** Correspondence between the five factors of CHIP-16 and the three factors of CHIP-45

CHIP-16	CHIP-45
	*Family integration, cooperation and an optimistic definition of the situation* (19 items)
Security and stability (2)	1. Communicating about personal feelings and concerns with my spouse
Security and stability (2)	3. Trusting my spouse (or former spouse) to help support me and my child
	6. Believing that my child will get better
	8. Showing that I am strong
	11. Taking good care of all medical equipment at home
	13. Getting other members of the family to help with chores and tasks at home
Belief and trust (5)	16. Believing that the medical center/hospital considers my family’s best interest
Belief and trust (5)	18. Believing in God
Home care (2)	21. Doing things together as a family (involving all members of the family)
	23. Believing that my child is getting the best medical care possible
Home care (2)	26. Doing things with family relatives
	28. Reminding myself that I have many things to be thankful for
Spouse and child relationship (4)	31. Encouraging my child with a medical condition to be more independent
Spouse and child relationship (4)	36. Building a closer relationship with my spouse
Spouse and child relationship (4)	38. Investing myself in my child
Family involvement (3)	41. Trying to maintain family stability
Family involvement (3)	43. Having my child with a medical condition seen at a clinic/hospital on a regular basis
	44. Believing that things will always work out
	45. Doing things with my children
	*Maintaining social support, self-esteem and psychological stability* (18 items)
	2. Engaging in relationships and friendships
	4. Sleeping
	7. Working in outside employment
	9. Purchasing gifts for myself and/or other family members
	12. Eating
	14. Getting away by myself
Belief and trust (5)	17. Building close relationships with people
	19. Developing myself as a person
	22. Investing time and energy in my job
	24. Entertaining friends at our home
	27. Becoming more self-reliant and independent
	29. Concentrating on hobbies (art, music, jogging, etc.)
	32. Keeping myself in shape and well groomed
	33. Engaging in social activities (parties, etc.) with friends
	34. Going out with my spouse on a regular basis
	37. Allowing myself to be angry
Family involvement (3)	39. Communicating with someone (not professional counselor/doctor) about how I feel
	42. Being able to get away from the home care tasks and responsibilities for some relief
	*Understanding the health care situation through communication with other parents and consultation with the health care-team* (8 items)
Belief and trust (5)	5. Communicating with the medical staff (nurses, social worker, etc.) when we visit the medical center
	10. Communicating with other individuals/parents in the same situation
Belief and trust (5)	15. Communicating with a doctor about my concerns about my child with a medical condition
	20. Communicating with other parents in the same type of situation and learning about their experiences
	25. Reading about how other persons in my situation addresses the situation
	30. Explaining family situation to friends and neighbors such that they can understand us
Spouse and child relationship (4)	35. Ensuring that the prescribed medical treatments for my child are carried out at home on a daily basis
	40. Reading more about the medical problem that concerns me

The number of items of each factor is indicated in parentheses

### Testing the five-factor model by a CFA

**Fig. 4:**
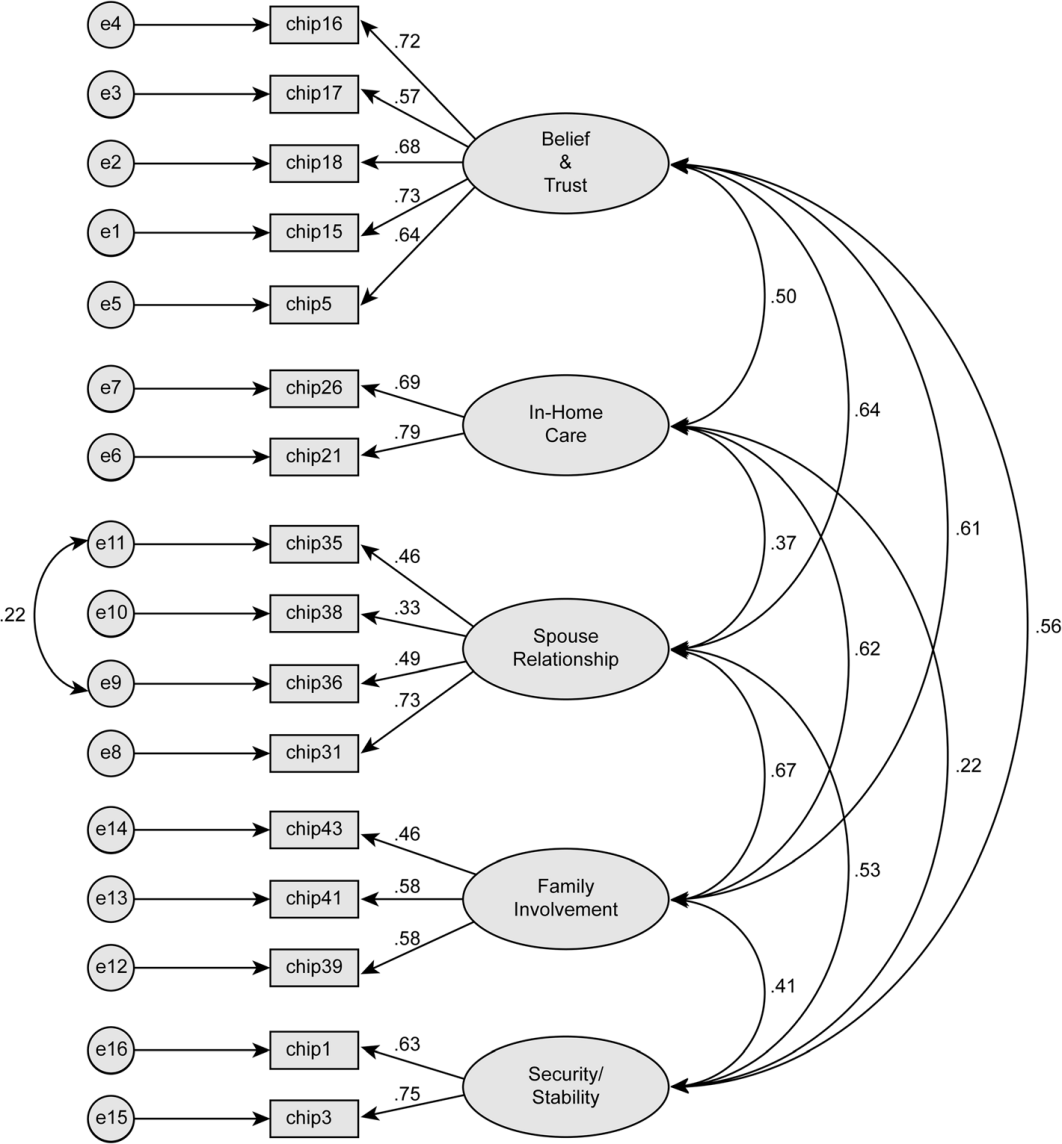
Five-factor model of the final 16 selected items in a subsample of 215 participants

A CFA was performed based on the subsample of 215 randomly selected respondents. The CFA results are presented in Fig. 4, and the fit indices are summarized in Table 1. The solution was admissible, and all parameters were significant. To improve the model fit, a parameter was freed, i.e., the correlation between two residual measurements (from items 35 and 36). The values of the fit indices ranged from acceptable (GFI, AGFI and IFI) to good (χ^2^/df and RMSEA), and the TWI value was close to .90. The parsimony was high because the parsimony ratio was greater than three quarters, and the values of the parsimonious indices (PNFI and PCFI) ranged between .60 and .80.

### Concurrent construct validity

The five-factor model of the CHIP-16 was subjected to three concurrent validity construct proofs involving BAI, BDI, and the sociodemographic variable sex. Scores above or below the cut-off value for the BAI defined the groups with or without anxiety (≥ 10 or < 10, respectively). Similarly, scores above or below the cut-off value for the BDI-II defined the groups with or without depression (≥ 14 or < 14, respectively).

The mean CHIP-16 score in the group without anxiety was higher than that in the group with anxiety (*t*[385] = 1.66, *p* < .05). Among the factors, the only significant difference between the groups with and without anxiety was the Spousal/partner relationship factor mean score (*t*[385] = 2.02, *p* < .05). The caregivers in the group without anxiety chose the coping pattern consisting of sharing worries and fears with their spouses, trusting and receiving spousal support more often than those in the group with anxiety. In both cases, the Cohen’s *d* values were above .20 but below .50; thus, the effect sizes were small. Although the mean CHIP-16 and its overall five-factor scores among the caregivers with depression were higher than those among the caregivers without depression, the statistical significance and effect sizes of these differences were trivial.

Comparing the mean scores of the total CHIP-16 and its five factors between the sexes, there was a significant difference in the home-care factor (*t*[464] = 2.35, *p* = .02; not assuming equality of variances: *F*[1, 401] = 9.33, *p* < .01). The mean of the women was higher than the mean of the men, and there was a small effect size of sex on the factor scores (Hedges’ *g* = 0.38, 95% CI: 0.14, 0.62). The women’s means were higher than the men’s means in the factors of spouse and child relationship and family involvement, but these differences were not significant.

## Discussion

To fulfill the first objective of this research, we obtained a translation of the CHIP adapted to the linguistic context of Mexico. The processes of reverse translation, review of discrepancies by experts in philology and health psychology, and corrections of difficulties in understanding the items offer good guarantees of compliance with the objectives.

The second objective was to test the correlated three-factor model proposed by McCubbin et al. [11]. Clearly, the factor model was inadequate. On the one hand, the model lacked goodness of fit. On the other hand, its three measurement models did not show convergent validity or discriminant validity when these properties were checked following the third objective. The differences in the cultural and clinical context between the present study and McCubbin’s studies may have driven the inadequacy of the factor model [47, 48], as is also indicated by studies performed in other countries [11]; thus, it was necessary to explore and validate a new model.

Regarding the fourth objective of this study related to the exploration of the CHIP factor structure, the model of correlated factors was reduced to 16 items to achieve a good-to-acceptable fit, which is better than the fit of the original model. The EFA with the 16 selected items strongly suggested five factors because the empirical criteria used to determine the number of factors converged to five factors, and high or very high factor loads were obtained. Therefore, this model seems to be appropriate for the data obtained from the Mexican sample of family caregivers of children with chronic diseases.

The original model [11] included a first factor with 19 items regarding maintaining family integration, cooperation, and an optimistic definition of the situation (items 1, 3, 6, 8, 11, 13, 16, 18, 21, 23, 26, 28, 31, 36, 38, 41,43,44, and 45). In light of the new model, this first original factor appears to be a general factor because it contains 11 of the 16 items of the new model, and these 11 items are distributed throughout the five new factors as follows: items 1 and 3 were moved to the security and stability factor; items 16 and 18 were moved to the beliefs and trust factor; items 21 and 26 were moved to the home-care factor; items 31, 36, and 38 were moved to the spouse/partner relationship factor; and items 41 and 43 were moved to the family involvement factor. The new five-factor structure suggests that coping can be associated with medical care, personal strength, perceived social support, the spouse, and family cohesion.

The second original factor of social support, self-esteem, and psychological stability [11], comprising18 indicators (items 2, 4, 7, 9, 12, 14, 17, 19, 22, 24, 27, 29, 32, 33, 34, 37, 39, and 42), was reduced to two indicators (items 17 and 39); one of the two items was moved to the new factor Beliefs and trust (item 17), and the other item was moved to the Family involvement factor (item 39). It appears that the coping inventory for parents suggests new dimensions for evaluating the affective links among family members, confidence in family caregivers, and the expression of feelings in coping with a child’s pediatric chronic disease.

The third original factor concerning understanding the medical situation through communication with other parents and consultation with medical personnel [11] originally comprised eight indicators (items 5, 10, 15, 20, 25, 30, 35, and 40) and was reduced to three indicators (items 5, 15, and 35). Two of these three items were moved to the beliefs and trust factor (items 5 and 15), and the third item was moved to the spouse/partner relationship factor (item 35). This result suggests that addressing parents of children with chronic diseases can be associated with beliefs and trust in the medical staff of a hospital.

The new five-factor model clearly accentuates the role of the family in coping with a child’s disease. The first factor in the US model regarding family integration and cooperation is explained by four factors (home care, the spouse/partner relationship, family involvement, and security and stability) and includes an item regarding belief in God. Previous studies comparing Mexican and American cultures have emphasized the importance of the family in the psychology of Mexicans [47, 49] and the importance of religion [48]. In particular, the importance of religion and family in the context of adaptation to a chronic disease has been highlighted in several studies conducted in Mexico [50, 51].

The internal consistency of the scale and its factors, the convergent validity of each factor, and the discriminant validity between the factors were favorable. In previous studies, the overall internal consistency of the original 45-item CHIP has usually been acceptable based on estimations using Cronbach’s alpha [29, 52–54], and good values of internal consistency have been reported for the overall scale [55] and even the overall scale and its first factor [56]. In the present study, similar to previous studies, the CHIP-16 overall reliability and the reliability of its first factor were good when evaluated using the McDonald coefficient omega, which is more appropriate for the estimation of internal consistency [38].

The internal consistency of the four remaining factors was acceptable and converged with the three-factor structure in previous studies [11, 29, 55]. Although the number of indicators of the five factors is low, which negatively affects the value of the internal consistency and renders reaching a high value difficult [57], McDonald’s coefficient omega allowed us to test for an acceptable internal consistency, resulting in a favorable outcome of the goodness of this index, which is still used minimally [38, 39]. The decision to retain such a small number of indicators was motivated by the need to meet the objective of obtaining a good data fit in an independent sample. This substantial reduction in items is supported by the convergence of the empirical criteria used to determine the number of factors and the internal consistency and convergence and discriminant validity of each factor.

Whether this reduction of items leads to a loss of validity in the contents of the CHIP-16 is a notable concern. Notably, the semantic range of the new model is quite large; the content of the three factors of the original model is qualified in a more differentiated manner with a minimum number of indicators; hence, no loss of content validity occurs despite the sharp reduction in items. Future studies may broaden the number of indicators of the factors, such as home care, family involvement, and stability and security, such that they reach four or five indicators similar to the other two factors. A qualitative methodology with focus groups could be used for this purpose [58].

The fifth objective of this research was to test the factor model derived from the EFA in an independent sample. The result was good to the extent that the EFA allowed for the identification of factors with clear discriminant and convergent validity. Consequently, this model was validated by fit indices ranging from good to acceptable. Similar to previous confirmatory studies [5, 59], the fit to the data improved after some items were eliminated.

Regarding the sixth objective, demonstrating the concurrent validity of the CHIP-16 with respect to anxiety and depression, poor overall coping and particularly poor coping through support in the marital relationship were observed in persons with anxiety compared with those who did not experience anxiety (those below the cut-off for the BAI) [30], and there was a small effect size of anxiety on coping. However, the groups with or without depression (those above or below the cut-off for the BDI-II [2, 32]) did not differ in terms of the total score or the five factors of the CHIP-16. In Mexico, factors are associated with anxiety in family caregivers of children with chronic diseases [60]. In Korean mothers, a relationship was identified between CHIP coping patterns and psychological distress and imbalance, suggesting the effect of anxiety [19]. A correlation between coping with diseases and anxiety was observed in 105 mothers and 21 fathers of children with cancer in the US using the Parenting Stress Index-Short Form [61]. Other previous studies have examined the relationship between coping patterns in mothers (measured using the CHIP) and anxiety and depression in their children [5, 62]. These studies identified relationships with small effect sizes but found stronger effects on anxiety than depression. Although the evidence of concurrent construct validity appears to be limited, our results are consistent with those of previous investigations.

In contrast, as further proof of the concurrent construct validity, it was expected that the average number of women would be greater in coping due to their seeking support from the family and the spouse, family involvement and home care [20, 21, 63]. The expected trend was observed in these factors, although a difference was observed only in home care. It appears that in situations that involve caring for a child, the coping strategies used by mothers and fathers are very similar.

Two limitations of this study are the use of a nonprobability sample and a limited sample size in the initial EFA. This study’s strengths include the fact that the exploratory and confirmatory factor analyses were conducted using independent samples and that the CFA had more than 10 cases per item.

## Conclusions

The CHIP-16 (reduced to 16 items) in the present sample of 405 Mexican parents and caregivers entrusted with the care of a chronically diseased child showed the following five-factor structure: beliefs/trust, spouse/partner relationship, home care, family involvement, and stability/security. Its overall internal consistency was good based on McDonald’s omega ordinal coefficient. The internal consistency of the belief and trust factor was also good, and that of its four remaining factors was acceptable. The five factors showed convergent and discriminant validity. In addition to item reduction, the model of five correlated factors showed acceptable indices and high parsimony. The average of the home-care factor was greater in women than in men, and anxiety was related to the CHIP-16 total score and the spouse/partner relationship factor. Similar to previous studies, the overall CHIP-16 score and the scores of the five factors were independent of depression, further providing evidence of the concurrent construct validity of the reduced version of the CHIP.

We suggest replicating this study using probability samples. In addition, attempts can be made to expand the number of indicators of each factor to four or five. Thus, focus groups [58] or natural semantic networks [64] can be used. The test-retest reliability, invariance across multiple time points and sexes, and content validity can be established in further research.

## Data Availability

The dataset supporting the conclusions of this publication is included with the article.

## Abbreviations

AVE: Average Variance Extracted
BAI: Beck Anxiety Inventory
BDI: Beck Depression Inventory
CHIP: Coping Health Inventory for Parents
CHIP-16: Coping Health Inventory Reduced to 16 Items
CFA: Confirmatory Factor Analysis
EFA: Exploratory Factor Analysis
